# BrajText-Saar: A structured cultural dataset for cultural text mining in indian heritage texts

**DOI:** 10.1016/j.dib.2026.113086

**Published:** 2026-07-16

**Authors:** Mukta M. Deshpande, Prafulla B. Bafna

**Affiliations:** Symbiosis Institute of Computer Studies and Research (SICSR), Symbiosis International (Deemed University) (SIU), Model Colony, Pune, Maharashtra, 411016, India

**Keywords:** Braj language, Indian regional language, Natural language processing (NLP), Cultural text mining, Data Pre-processing, Noise removal

## Abstract

India is one of the countries where many regional languages are spoken in different parts of the country. Each part of the country has its unique cultural traditions; therefore, an abundance of cultural texts is available in Indian Regional languages. The cultural texts are mostly ancient scriptures that represent the community's heritage, values, and ancient knowledge. Braj is one of the Indian regional languages that represents the cultural heritage of the Braj region. The BrajText-Saar dataset contains texts from the Braj language, which features a variety of prehistoric scripts. The data was collected from the Maan Mandir trust's portal, which operates to maintain Braj culture and heritage. The Braj text primarily explains the divine actions and the life of Lord Krishna in the form of poetry and devotional songs. Offline Braj literature from renowned writers is available in manuscript form. This represents an opportunity for cultural text mining in the Braj Language. The developed dataset opens new paths in various research areas, including language studies, sentiment analysis, and emotion analysis, and is also suitable for digital humanities research.

Specifications Table

**Table:** 

Subject	Computer Sciences
Specific subject area	Cultural Text Mining, Indian Regional Language Processing
Type of data	Text file (.Txt)
Data collection	Downloaded from Download Maan Mandir Publications (Books) FREE | Maan Mandir Seva Sansthan Trust, Evaluated by Native language experts
Data source location	Braj Region, Uttar Pradesh (India), website Download Maan Mandir Publications (Books) FREE | Maan Mandir Seva Sansthan Trust
Data accessibility	Repository name: BrajText-Saar Dataset Data identification number: *(*10.17632/pg624k2rky.1*)* Direct URL to data: BrajText-Saar - Mendeley Data Instructions for accessing these data: The Data Can be downloaded by using the above link
Related research article	None

## 1 Value of the Data

• Existing Indic corpora, such as IndicNLP, mainly focus on multilingual or major high-resource Indian regional languages. In contrast, the developed dataset targets Braj, a low-resource language, and provides a curated, pre-processed corpus. A customised pre-processing pipeline is applied to capture language-specific features. These characteristics distinguish it from general Indic datasets and improve its suitability for NLP tasks.
• This dataset will be a valuable resource in digital humanities for conducting research in Philosophy and history.
• This dataset will be helpful in language research to analyze the semantics, grammar, and morphology of the Braj language.
• It will also help researchers to understand cultural values, emotions, and sentiments in Braj heritage texts.

## 2 Background

Presently available datasets of Indian languages, mainly with an emphasis on modern linguistic texts, but often overlook cultural texts. They also neglect many dialects, which restricts the ability to study cultural texts. The Braj language is one of the underrepresented Indian regional languages. Many NLP systems need extensive data and language expertise. However, both are costly and require language expertise [1]. The Indian government, through projects like LDC-IL [2], has developed resources for many Indian regional languages. AI4Bharat has developed the IndicNLP repository of texts to support many Indian regional languages [3]. It covers 10 Indian languages from two groups. This increases resources for NLP work. Alongside these collections, related work [4] demonstrates a system that utilizes natural language tools to scan social media for instances of cyberbullying. Also, [5] discusses the creation of text groups for Indian languages that lack sufficient data. These efforts show that more NLP resources are being made. They also demonstrate how NLP tools enable computers to handle spoken and written language. This allows people to automate more tasks. The Braj language is significant to both history and religion, yet it remains understudied in textual research. Recent work has begun to address this issue by creating and labeling data groups, using data methods, and verifying the results. The primary goal is to focus on Indian languages for which limited data is available [[6], [7], [8]]. Sentiment analysis is now a primary focus for studying people's feelings expressed in text [9]. Named entity recognition (NER) helps identify key information from raw text. Named Entity Recognition (NER) is a popular NLP technique that classifies entities from texts [10]. Stopword removal is one of the text pre-processing steps, which eliminates words from texts that carry little meaning within the text [11]. The data pre-processing steps reduce the data size and also remove noise from the text [12]. Sentiment analysis is conducted using various data pre-processing steps on the Manipuri language, a regional language in India [13].

Text summarizing is one of the NLP strategies that condenses lengthy texts while preserving important information [14]. [15] created a Hindi dataset to do sentiment analysis on product evaluations. The process of obtaining information from various sources is known as data collection, and it is often difficult due to its complexity. It also needs careful handling of the collected data [16]. For developing the Marathi dataset for the sentiment analysis task [17], challenges arise. All of the above research was conducted for Indian regional languages, highlighting their work and challenges. This ensures that the BrajText-Saar dataset will be domain-specific, carefully curated, and a systematically pre-processed dataset developed to support NLP tasks such as sentiment and emotion analysis. It can also be useful in language and digital humanities research [18].

## 3 Data Description

This section provides detailed information about the dataset's size, sample records, and file formats for a better understanding of the dataset.

### 3.1 Overview of the dataset

This dataset contains Braj language devotional and cultural texts, including poetry. Its classical format blends kindness and devotion, making it ideal for Braj cultural text mining. The dataset has the processed BrajText-Saar file and the Braj_Stopwords_N_S_List, a selected UTF-8 encoded file containing a collection of stopwords, memorable characters, numbers, and high-frequency auxiliary words commonly removed to enhance text mining. Both files are supplied in .txt format.

### 3.2 Dataset description

Table 1 presents statistics for BrajText-Saar, including token counts before and after pre-processing, as well as the number of stopwords, special characters, and punctuation. It also includes the number of tokens removed, with their total percentage and the file formats of the datasets. However, the pre-processed dataset is moderate in size, highly curated, and supported by lexical diversity measures suitable for various NLP tasks, such as feature extraction and sentiment analysis, in low-resource contexts like Braj. The availability of a pre-processed dataset is often more challenging than that of a large-scale dataset. The dataset is well-suited for tasks such as clustering, sentiment analysis, and pattern mining in Braj Text due to its linguistic diversity and cultural significance. This dataset is primarily intended for implementing text mining tasks, such as sentiment analysis, clustering, and feature extraction. It can also be used for exploratory language analysis. This dataset is suitable for implementing machine learning techniques, but insufficient for training deep learning models due to its moderate size. This dataset does not contain sentence boundaries, thematic labels, or any metadata.

**Table 1:** Dataset Statistics.

Elements	Particulars
Tokens (words) before pre-processing	17, 372
After Pre-processing Tokens(words)	14,741
Unique Tokens	4508
Identified Stopwords	44
Identified special characters	15
Identified Numbers	3
Number of tokens (words) Removed	2631
Percentage of Tokens Removed	15.1%
Datasets File Formats	.txt

### 3.3 Sample records from brajtext-saar

**Fig. 1:**
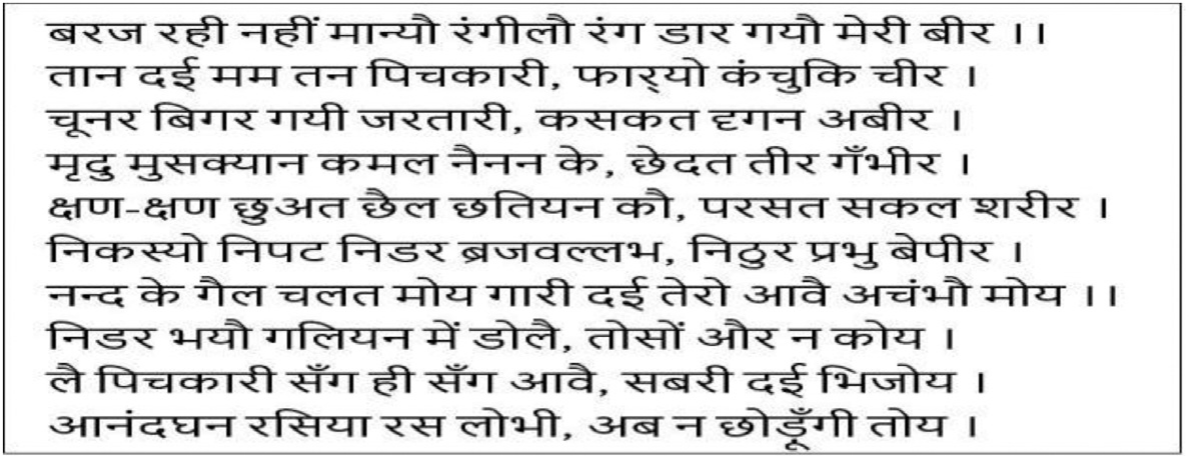
Sample Text before pre-processing.

**Fig. 2:**
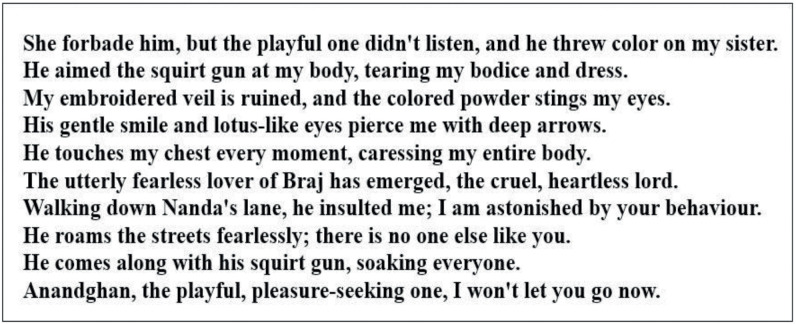
English Translation of Sample Text.

Fig. 1 shows the sample text with stopwords prior to pre-processing, while Fig. 2 shows its English translation prior to pre-processing.

**Fig. 3:**
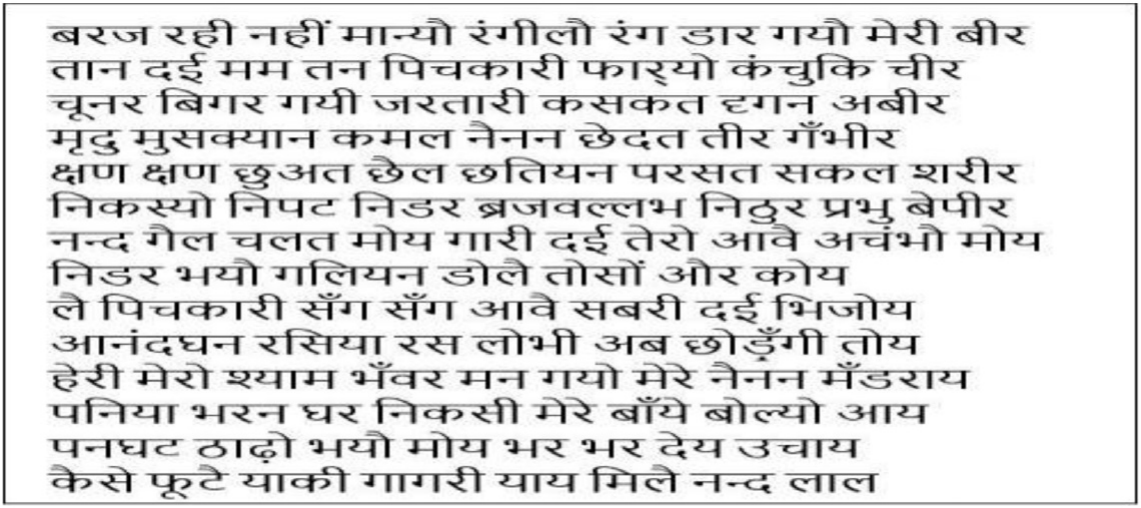
Sample pre-processed text.

Fig. 3 shows a sample pre-processed text. The pre-processing step reduced the word count by eliminating words with less meaning. making the text more representative of meaningful words.

## 4 Experimental Design, Materials, and Methods

This section describes the techniques implemented and various experiments carried out for the development of the dataset.

**Fig. 4:**
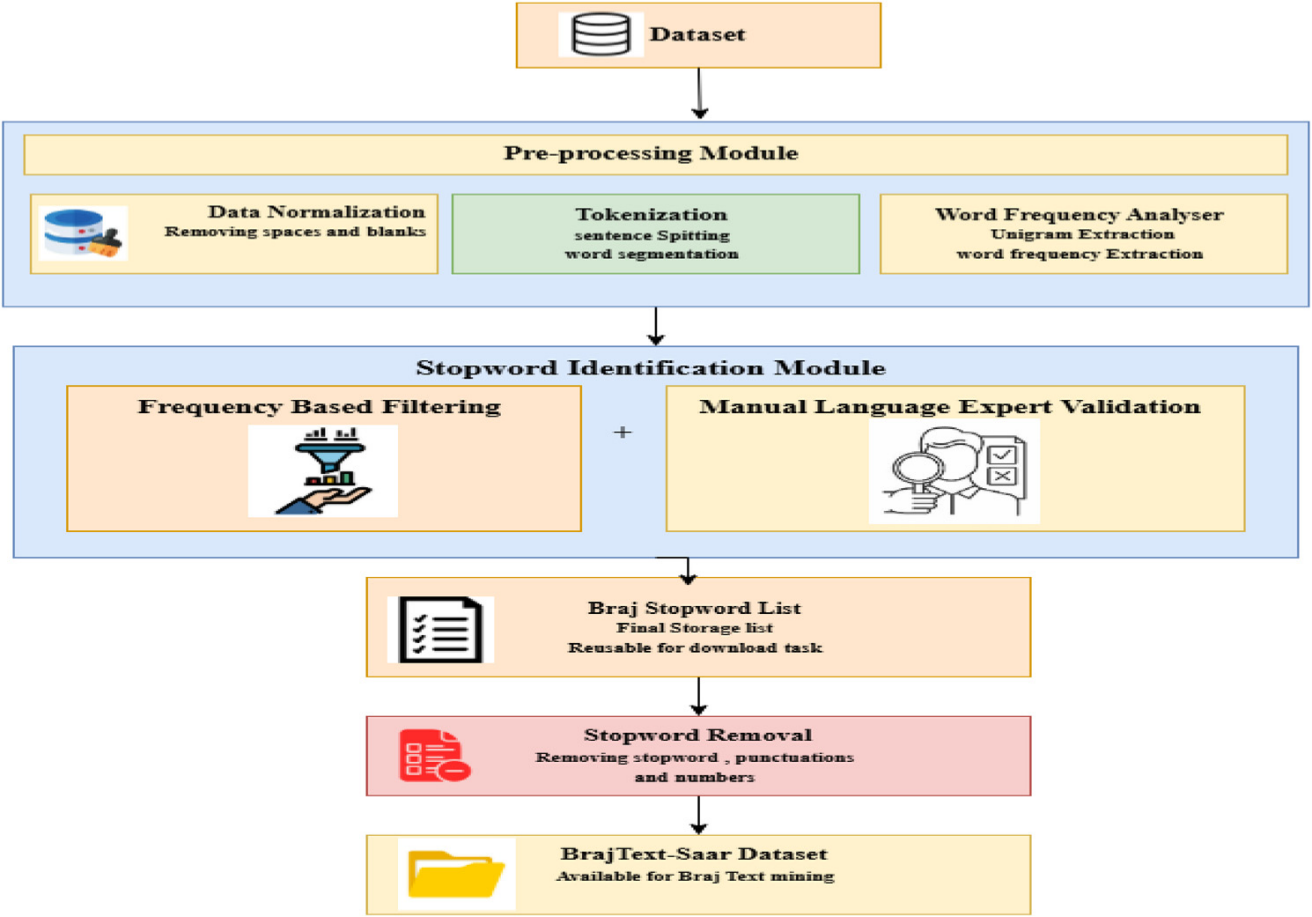
Architecture Diagram for the Development of the Dataset.

The architectural diagram for developing the BrajText-Saar dataset is shown in Fig. 4. It describes several steps to prepare the text for further mining.

### 4.1 Data collection process

The text was collected from the Man Mandir organization website in February 2025. The data was saved in a text file using UTF-8 encoding as RawBraj.txt.

### 4.2 Text normalization

Text normalization (NFC) was performed to uphold text consistency. It also eliminates blank lines and whitespace. The Devanagari symbol ("।") symbolizes sentence boundaries. Each sentence was drafted on a separate line. There was no need for extended pre-processing such as regex, diacritic, and orthographic normalization due to the standard form of the dataset

All normalized text was saved in UTF-8 encoding for uniformity and reuse. Data normalization is mathematically described in Eq. (1).

(1) $$C^{\prime }=C-Spaces\;-\;trim\left(C\right)\;-\;blank\;lines$$

### 4.3 Tokenization

Tokenization is an essential process in natural language processing that divides text into smaller units, known as tokens.

**Fig. 5:**
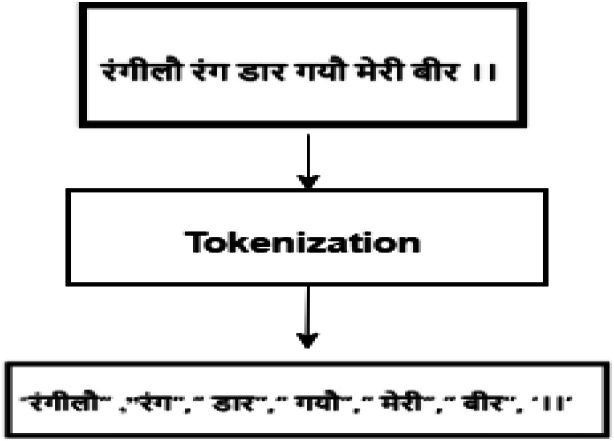
Tokenization of the sample word.

Fig. 5 illustrates the tokenization process demonstrated on a sample sentence. Tokenization splits a sentence into individual words, known as tokens.

Table 2 illustrates the tokenization process applied to sample Braj sentences.

**Table 2:** Tokenization sample for BrajText-Saar dataset.

Sr. No	Sample Braj Sentence	Tokenization	Sentence Translation in English	Tokenization Translations In English
1	बरज रही नहीं मान्यौ	“बरज”,” रही”,” नहीं “, “मान्यौ”	I am not getting angry	“I”,” am”,”not”, “getting”, “ angry”
2	रंगीलौ रंग डार गयौ मेरी बीर ।।	“रंगीलौ”,”रंग”,” डार”,” गयौ”,” मेरी”,” बीर”, ‘।।‘	You have coloured me, my brave man	“You”,“have”,”coloured “, “me”, “my”, “brave”, “ man”
3	मोतिन माल गले सों तोरी, लहँगा फरिया रंग में बोरी ।	“मोतिन”, “ माल”,” गले”, “सों “, “तोरी”,”लहँगा “फरिया”, “ रंग”, “ में”, “बोरी”, “।“	You have pearl necklace around your neck,the lehenga is full of colour	“You”, “ have”, “ pearl”, “necklace”, “ around”, “your”, “neck”,”the”, “ lehenga”, “is”, “full”, “of”, “ colour”

The Mathematical Expression for the Tokenization process is defined in Eq. (2)

(2) $$T=Tokenize\left(C^{\prime }\right)\;$$

### 4.4 Stopword list preparation

#### 4.4.1 Frequency-based candidate stopword identification

The frequency count for every word was generated from the Braj text to identify tokens that appear with high frequency and low semantic meaning. Stopwords were determined using a two-step hybrid approach. The first is the statistical N-gram method, and the second is the help of Braj language experts. The statistical N-gram method identifies the most frequent words in a corpus. An N-gram identifies a sequence of N consecutive words. For example, in a unigram (N = 1), bigram (N = 2), or trigram (N = 3). In this study, unigrams (N = 1) are extracted from the corpus.

**Fig. 6:**
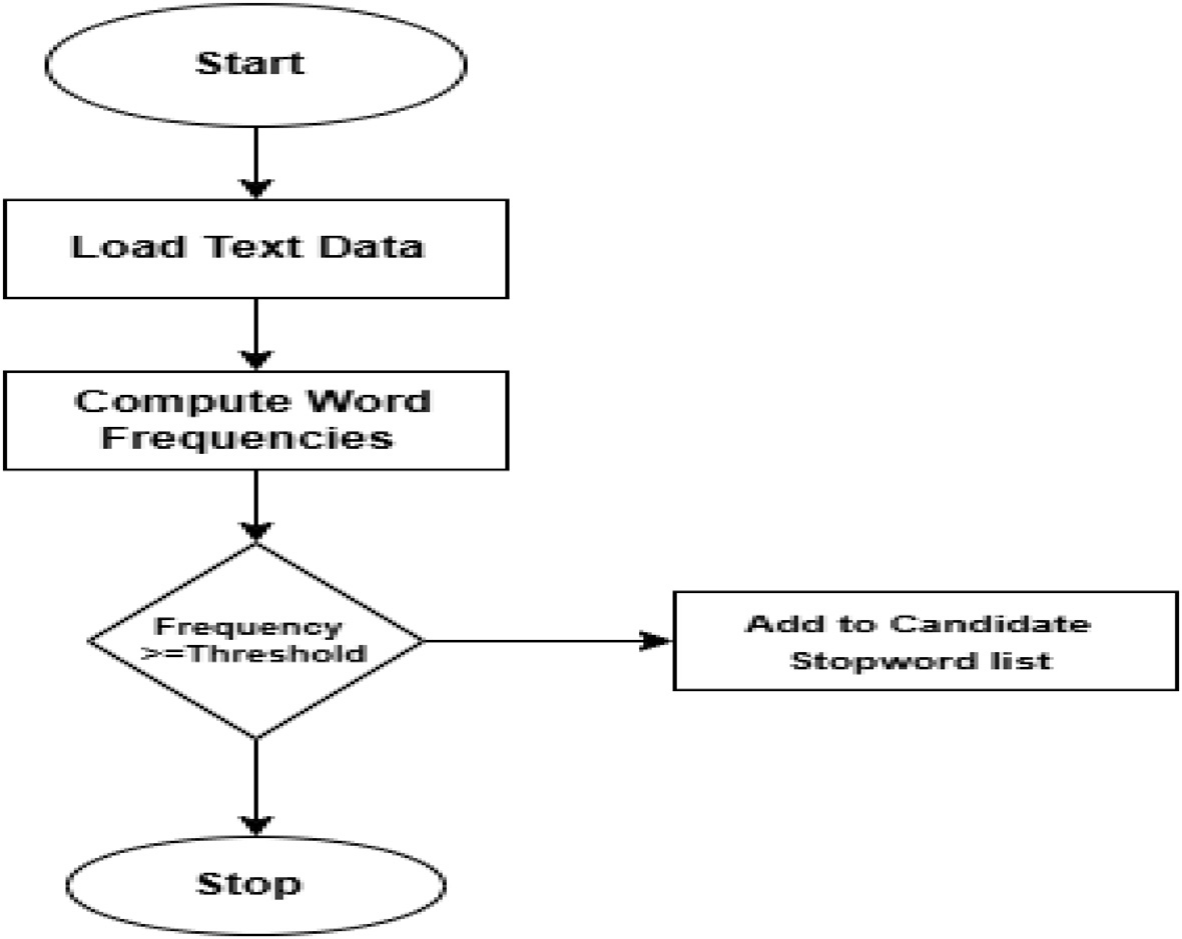
Frequency and threshold-based Stopword generation.

Fig. 6 illustrates the step-by-step process for generating a candidate Stopword list based on word frequency and threshold.

#### 4.4.2 Expert review and final stopword list

**Selection criterion**: The word frequency for each word is recorded. The threshold values collected from eight local language specialists are ɵ = {148, 146, 147, 150, 142, 144, 146, 144}. The mean (µ) is calculated as 145. To evaluate the local language specialist’s threshold recommendations, measures of dispersion, such as standard deviation and variance, are used.

The variance (σ^2^) and standard deviation (σ) were calculated for the mean value (µ) = 145, which comes as 5.61 and 2.3, respectively. These statistical measures confirm strong agreement among local language specialists' inputs.

The mean of 145 was approved as the average value with the help of eight local language specialists. All tokens appearing 145 times or more were selected as final stopwords. Table 3 shows that commonly used functional words such as “में” (in), “की” (belonging to), and “रे” (“o boy”) appear with a high frequency but contribute negligible semantic significance. These have been chosen for removal as a component of the stopword list. Important cultural and thematic words such as “कन्हैया” (Krishna), “आज” (today), and “राधा” (Radha) have been retained as they serve as crucial for understanding the context of Braj literature and devotional literature. Stopwords and their frequencies are identified.

**Table 3:** Sample words with their frequency from BrajText-Saar.

Sr.no	Word (Token)	Frequency	Translation (English)	Remark
1	“सों”	215	sou	Frequent word, Stopword
2	“कन्हैया”	4	Lord Krishna	Noun, Retained
3	“आज”	48	Today	Retained
4	“रे”	188	Vocative particle “O.”	Frequent word, Stopword
5	“राधा”	24	Radha	Noun, Retained

Braj language specialists then reviewed the shortlisted tokens to ensure that culturally meaningful, context-specific words were excluded from the list. They also included Special characters, numbers, and punctuation in the final stopword list. After revision, the final Braj Stopword list consisted of forty-four high-frequency stopwords, fifteen special characters, and three numbers.

**Fig. 7:**
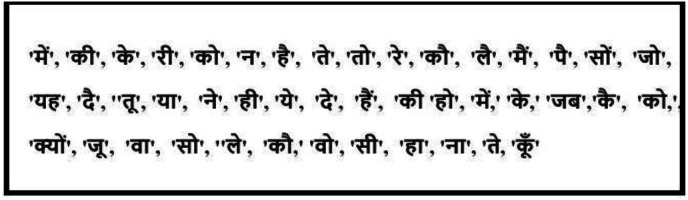
Stopword list.

Fig. 7 illustrates the Stopword list developed in this study, which was further used for pre-processing the data.

**Fig. 8:**
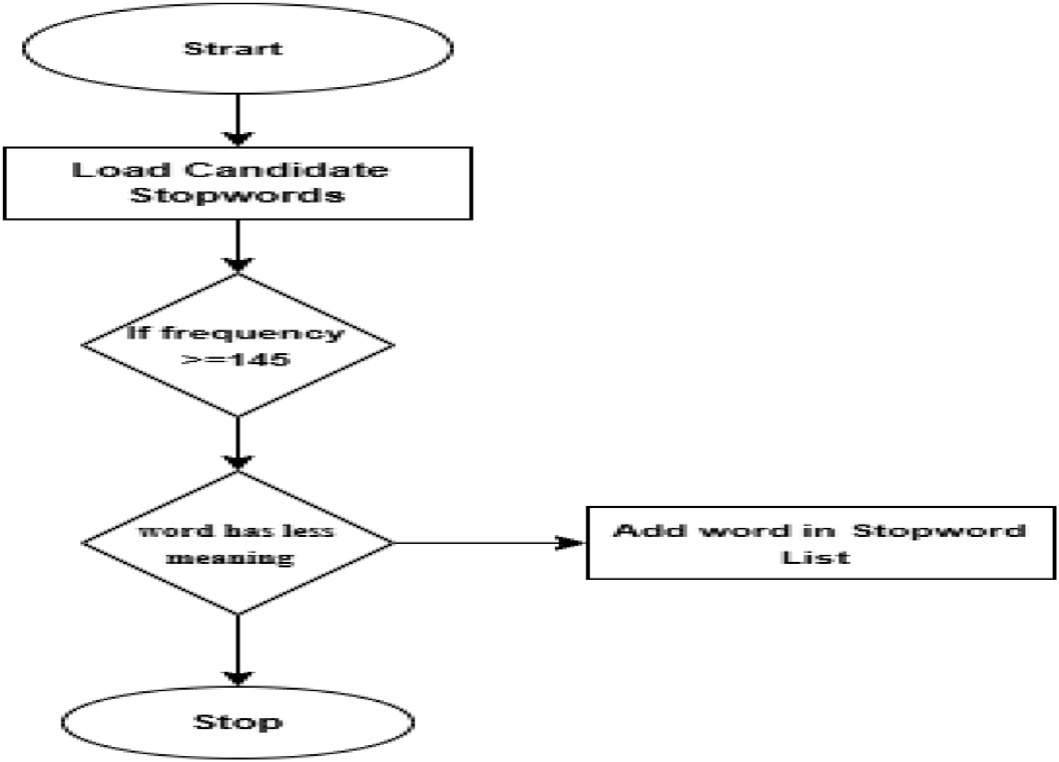
Stopword list generation with Expert Review.

Fig. 8 illustrates the final stopword generation process with the help of native language expert review and word importance in text.

Table 4 presents the sample sentences and their English translations from the cleaned dataset.

**Table 4:** Sample sentence with its English Translation after pre-processing.

Braj Text after (Stopword Removal)	Poetic English Translation
रंगीलौ रंग डार गयौ मेरी बीर	You have coloured me, my brave man.
मोतिन माल गले तोरी लहँगा फरिया रंग बोरी	You have pearl necklace around your neck, the lehenga is full of colour

After data pre-processing, the text is reduced in size compared to the original, retaining only the meaningful words from the dataset.

### 4.5 Evaluation and validation

The proposed dataset’s linguistic quality and lexical diversity are measured using TTR (Type-Token Ratio). The TTR is measured using the following Eq. (3).

(3) TTR=NumberofUniqueTokens/TotalNumberofTokens

TTR=4508/14741=0.3

**Fig. 9:**
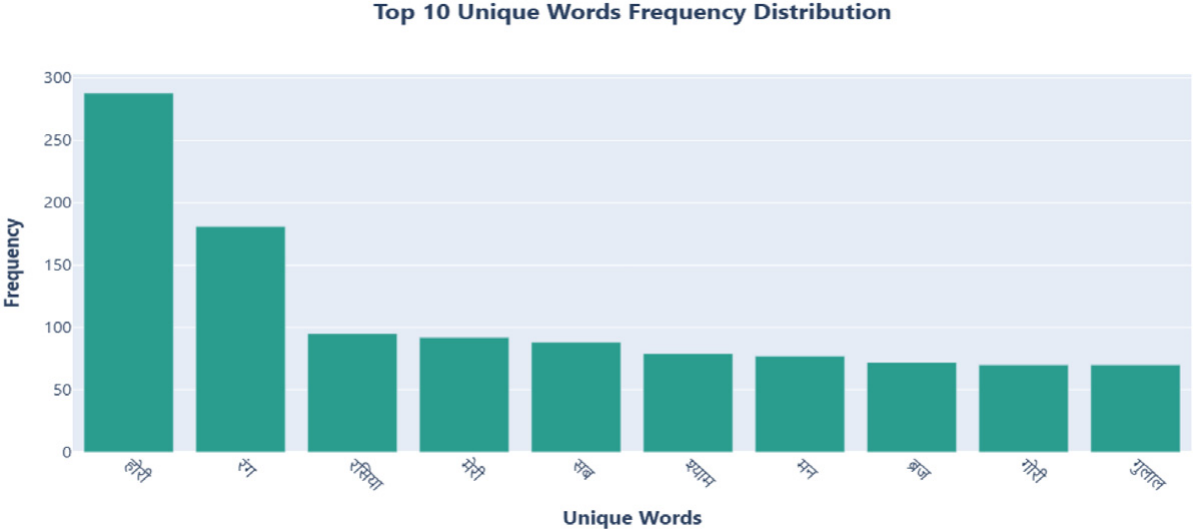
Top 10 unique words frequency distribution.

The proposed dataset with a TTR value of 0.3 indicates good lexical diversity and vocabulary, and it preserves language features. Fig. 9 illustrates the top 10 unique words frequency distribution for the analysis of the top 10 unique words from the dataset, with their respective frequency .

### 4.6 Software and reproducibility protocol

The Braj Stopword identification method follows a reproducible protocol. It follows steps such as tokenization, token frequency computation, expert local language-based threshold selection, and its statistical validation. Python 3.9, with its key libraries such as Pandas 2.3, which handles datasets, and Matplotlib 3.4 for visualization, was used in the Google Colab environment. IndicNLP library is utilized for data pre-processing. The local language specialists utilized Excel 2021 for evaluating stopwords.

**Fig. 10:**
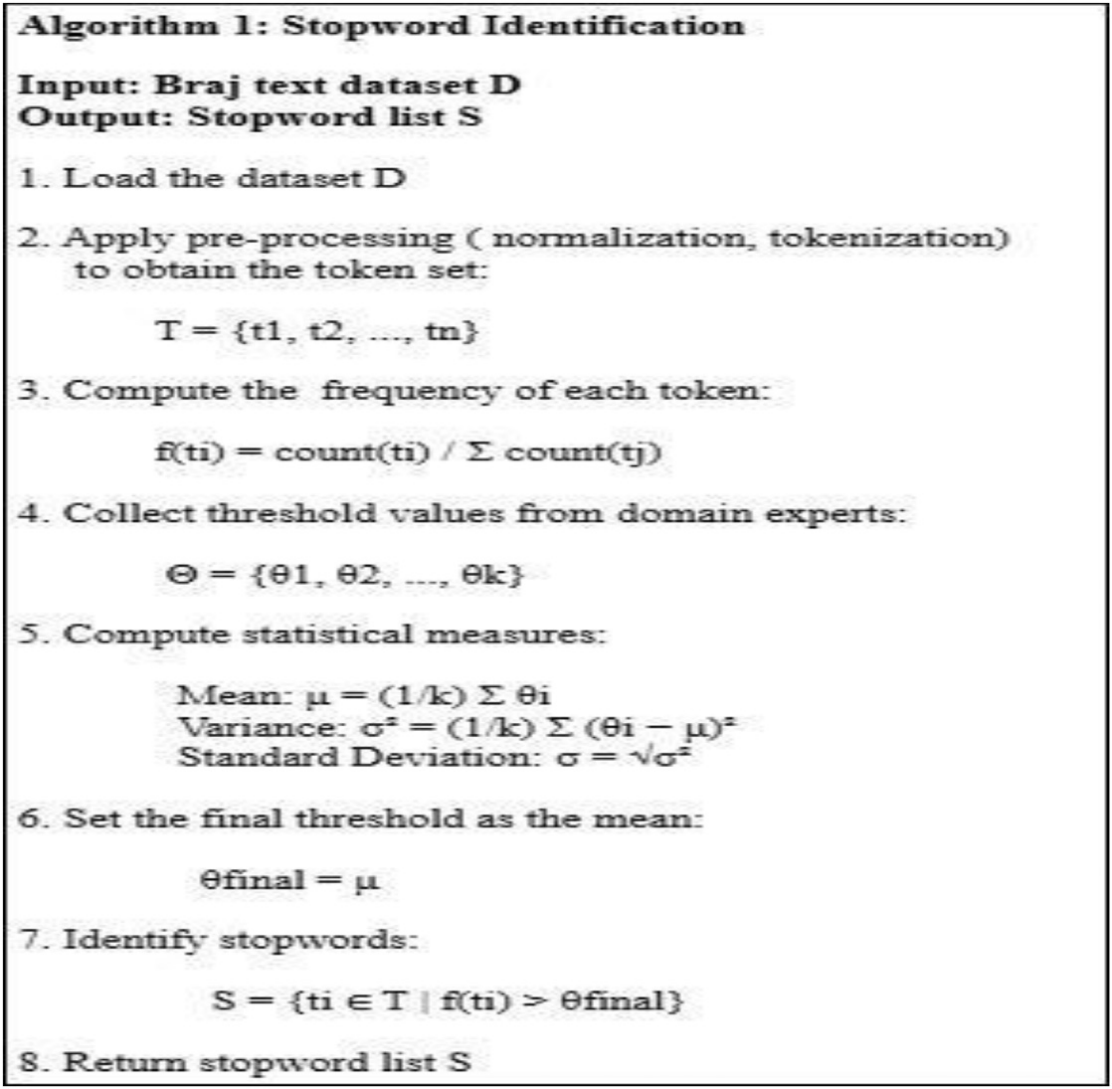
Algorithm for Braj Stopword Identification.

Fig. 10. illustrates an algorithm for Braj stopwords identification to pre-process the Braj Dataset and ensure clear reproducibility.

## Limitations

The BrajText-Saar mainly consists of devotional texts obtained from the Man Mandir Seva Santhan's website. Therefore, the language patterns captured are specific to cultural contexts. The Braj language lacks standardized tools such as stemmers and Part-of-speech taggers, which need manual intervention throughout pre-processing and Stopword identification.

## Ethics Statement

We certify that no human subjects nor animal experiments are used in the current work, and we have read and followed the ethical criteria for publication in Data in Brief. or any information gathered from social media sites. Furthermore, the data obtained from the source Man Mandir organization's website is publicly available and not subject to any restrictions or licensing requirements for copying, editing, or reuse.

The dataset is shared only for research purposes, and no original or copyrighted content is redistributed.

## Credit Author Statement

**Mukta M. Deshpande** Methodology, Conceptualization, Data curation, writing original draft; **Dr. Prafulla B. Bafna** Supervision and Reviewing; and Validation.

## Data and Code Availability

The dataset developed in this study is now publicly available. Please find the link here: https://data.mendeley.com/datasets/pg624k2rky/1.

The code to develop this dataset is available at: https://colab.research.google.com/drive/1Bt9wBmv4o-fjq0lm8JZrTnaOn-yMhuH

## Data Availability

Mendeley DataBrajText-Saar (Original data)

## References

[1] Aggarwal S., Kumar S., Mamidi R. (2021). Proceedings of the International Conference on Recent Advances in Natural Language Processing (RANLP 2021).

[2] Choudhary N. (2021). LDC-IL: the Indian repository of resources for language technology. Lang. Resour. Eval..

[3] Kunchukuttan, A., Mehta, P., et al. (2020). The AI4Bharat-IndicNLP corpus: monolingual corpora and word embeddings for indic languages.

[4] Valarmathi C., Velu A., Prasanth A., Dhanaraj R.K. (2025). 2025, the 4th International Conference on Computing and Information Technology (ICCIT).

[5] Dongare P., Jha G.N., Sobha L. (2024). Proceedings of the Seventh Workshop on Indian Language Data: Resources and Evaluation (WILDRE-7).

[6] Nalini M., Dhanraj R.K., Balusamy B., Abirami V., Kavya K. (2024). Hyperautomation For Next-Generation Industries.

[7] Harish B.S., Rangan R.K. (2020). A comprehensive survey on Indian regional language processing. SN Appl. Sci..

[8] Gupta M.K., Chandra P. (2020). A comprehensive survey of data mining. Int. J. Inf. Technol..

[9] Rakshitha K., Ramalingam H.M., Pavithra M., Advi H.D., Hegde M. (2021). Sentimental analysis of Indian regional languages on social media. Glob. Transit. Proc..

[10] Sharma R., Morwal S., Agarwal B. (2022). Named entity recognition using a neural language model and CRF for the Hindi language. Comput. Speech Lang..

[11] Rani R., Lobiyal D.K. (2018). Automatic construction of a generic stop word list for Hindi text. Procedia Comput. Sci..

[12] Rahimi Z., Homayounpour M.M. (2023). The impact of preprocessing on word embedding quality: a comparative study. Lang. Resour. Eval..

[13] Meetei L.S., Padma M., Ravi V. (2021). Low-resource language-specific pre-processing and features for sentiment analysis tasks. Lang. Resour. Eval..

[14] Thapa S., Adhikari S., Mishra S. (2021, March). Proceedings of 3rd International Conference on Computing Informatics and Networks: ICCIN 2020.

[15] Akhtar S.S., Ekbal A., Bhattacharyya P. (2016). Aspect-based sentiment analysis in Hindi: resource creation and evaluation. Cogn. Comput..

[16] Yennam S., Kalyani B.J.D., Pathakota S.S., Nookala S. (2024). In the 2024 International Conference on Recent Advances in Science and Engineering Technology (ICRASET).

[17] Sirsat S., Zulpe N. (2023, March). 2023, Somaiya International Conference on Technology and Information Management (SICTIM).

[18] Deshpande M., Bafna P. (2025).

